# Supplementary material for: Associations of socioeconomic disparities with buccal DNA-methylation measures of biological aging
Source: Clin Epigenetics. 2023 Apr 28;15:70. doi: 10.1186/s13148-023-01489-7 (PMC10148429; doi:10.1186/s13148-023-01489-7)
Supplement: Supplementary file 1 — Additional file 1: Fig. S1. Correlation matrix of socioeconomic variables with buccal DNA-methylation algorithms in full sample, excluding self-reported smokers, and residualizing DNAm algorithms for body mass index. In all plots, DNAm measures were residualized for chronological age. BMI was age- and sex-normed. [file 13148_2023_1489_MOESM1_ESM.docx]

# Supplemental Results

## Supplemental Figures

| (A) Full sample (n=1058) | (B) Excluding self-reported smokers (n=971) |
| --- | --- |
| 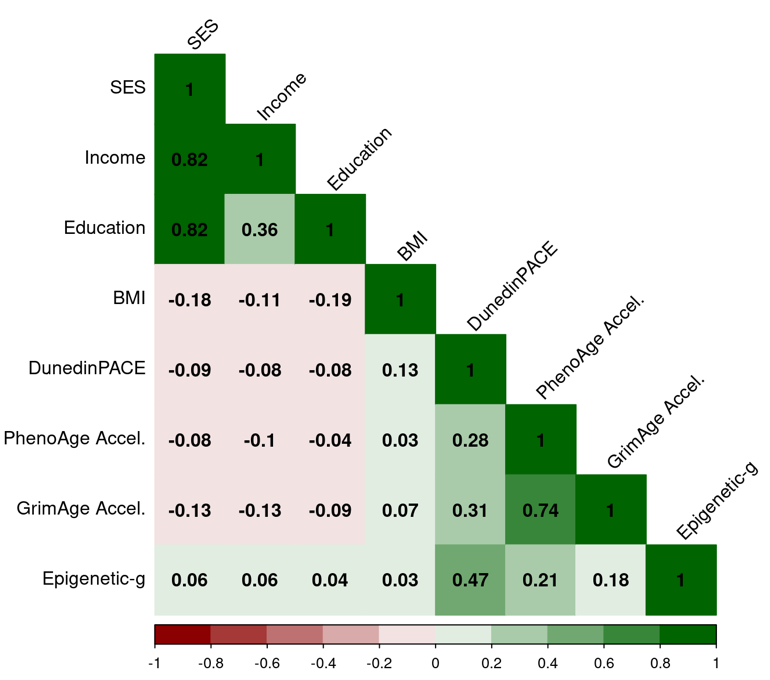 | 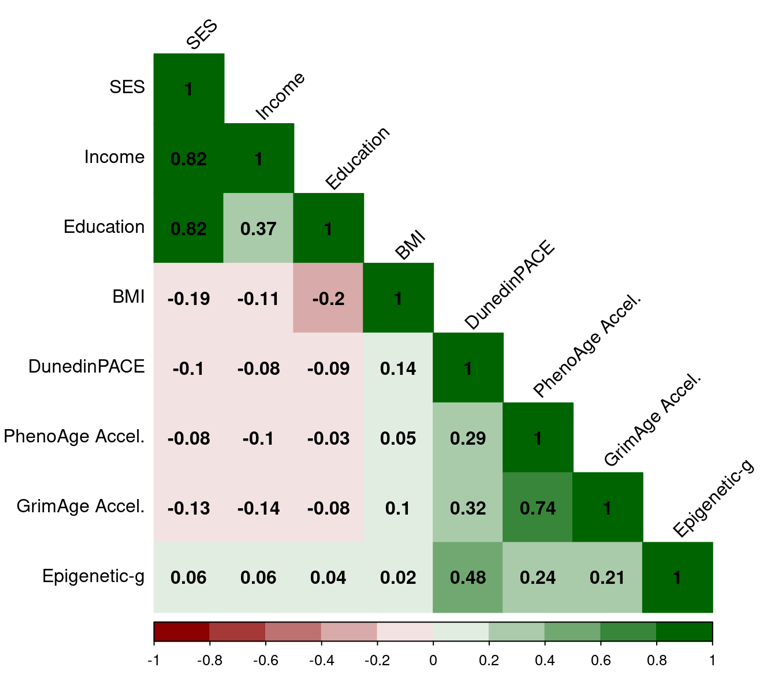 |
| (C) DNAm algorithms residualized for body mass index (n= 876)  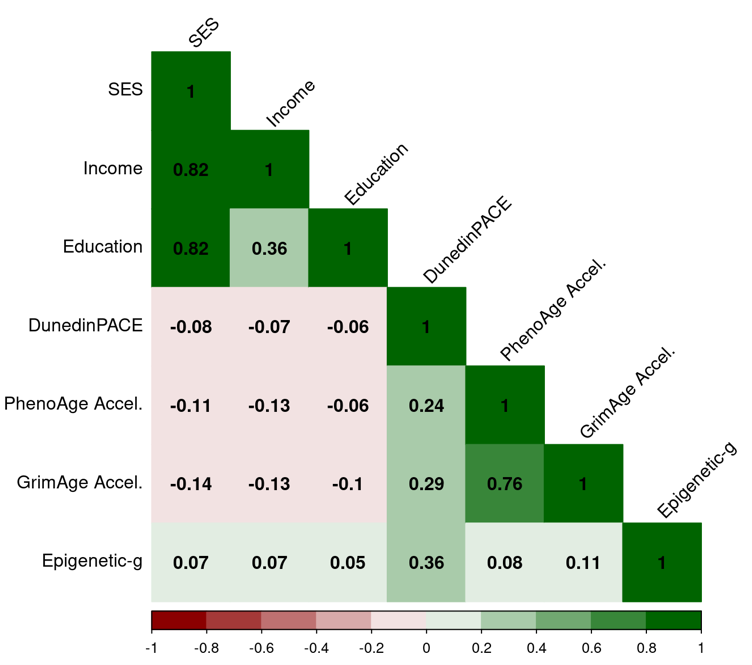 |  |
| **Supplementary Figure S1**. Correlation matrix of of socioeconomic variables with buccal DNA-methylation (DNAm) algorithms in (A) full sample, (B) excluding self-reported smokers, and (C) residualizing DNAm algorithms for body mass index (BMI). In all plots DNAm measures were residualized for chronological age. BMI was age- and sex-normed. | |
